# Editorial: Covid, Long Covid, Mental Health, Schools and Masks: how and why we failed child health communication during a pandemic

**DOI:** 10.3389/fped.2023.1104518

**Published:** 2023-02-08

**Authors:** Anna Camporesi, Vanessa Soares Lanziotti, Yonca Bulut, Deanna Behrens, Danilo Buonsenso

**Affiliations:** ^1^ Pediatric Anesthesia and Intensive Care, Vittore Buzzi Children's Hospital, Milano, Italy; ^2^ Pediatric Intensive Care Unit, Institute of Pediatrics, Federal University of Rio de Janeiro, Rio de Janeiro, Brazil; ^3^ Pediatric Intensive Care, UCLA Mattel Children's Hospital, Los Angeles, CA, United States; ^4^ Department of Woman and Child Health and Public Health, Fondazione Policlinico Universitario A. Gemelli IRCCS, Rome, Italy; ^5^ Centro di Salute Globale, Università Cattolica del Sacro Cuore, Roma, Italy

**Keywords:** COVID, long covid, children, masks, SARS-CoV-2

**Editorial on the Research Topic** The Global Burden of COVID-19 on Children’s Health

As more than 2 years have passed since the beginning of the pandemic, the time has come for the consideration and analysis of the achievements and advances in the field of child health. Such considerations are needed to better understand the best ways of managing the next pandemic.

In terms of medical achievements, this pandemic will be remembered for the historically rapid development of trials, vaccines, and drugs that have saved millions of lives. However, results are more controversial in terms of public health responses, communication, public opinion, and, overall, in terms of balancing actions that take into account the wider concept of well-being not limited to the recovery from illness.

In this regard, children are in a unique position to allow us to analyze the impact of the pandemic from a child-centered perspective. This is possible because, on one hand, the burden of acute COVID-19 has been clearly less significant in children (1), but on the other hand, children are intrinsically more fragile and often overlooked politically.

These peculiarities have led to the birth of two opposing extremist positions that, in our opinion, have ultimately hampered the societal approach to defending children's needs during the pandemic.

Let us start from the beginning: SARS-CoV-2 infection. Since the first data emerged in China, COVID-19 has led to hundreds of hospitalizations and deaths in adults worldwide. This, along with rigorous early lockdowns, led to observations of very low numbers of pediatric cases during the first wave. However, pediatric deaths or intensive care unit hospitalizations were registered during the very first months of the pandemic and described in early publications from China, the US, and Europe (2). Nevertheless, polarized messages to the public led to the misconception that COVID-19 did not affect children. These incorrect messages affected and still affect the societal response to the pandemic, including influencing scientists and parents in the decision of whether or not to vaccinate children (3). These messages became even more extreme with the much misused or overused observation that most lethal cases occurred in children with comorbidities. To our knowledge, never in history has this concept been stressed so heavily before, creating a sort of stigmatization of children affected by pre-existing comorbidities. Moreover, children can develop Multisystem Inflammatory Syndrome, which often requires intensive care unit admission and can even be fatal, a complication still poorly known to parents.

Such extreme opinions frequently led scientists to compare disease severities in unhelpful attempts to show which disease was more severe and therefore more important for pediatric health. In the media, this approach was not based on solid evidence but rather aimed at supporting a predefined position. A very common comparison was between respiratory syncytial virus (RSV), influenza, and COVID-19, highlighting the higher hospitalizations during RSV seasons or the more cardiovascular involvement of COVID-19. This approach is disrespectful toward all those families that have lost children to each of these conditions, or have disabled children from these viruses. A personalized risk approach is still far from routine practice and, unfortunately, every single pathogen has the potential to cause severe illness.

**Figure 1.**
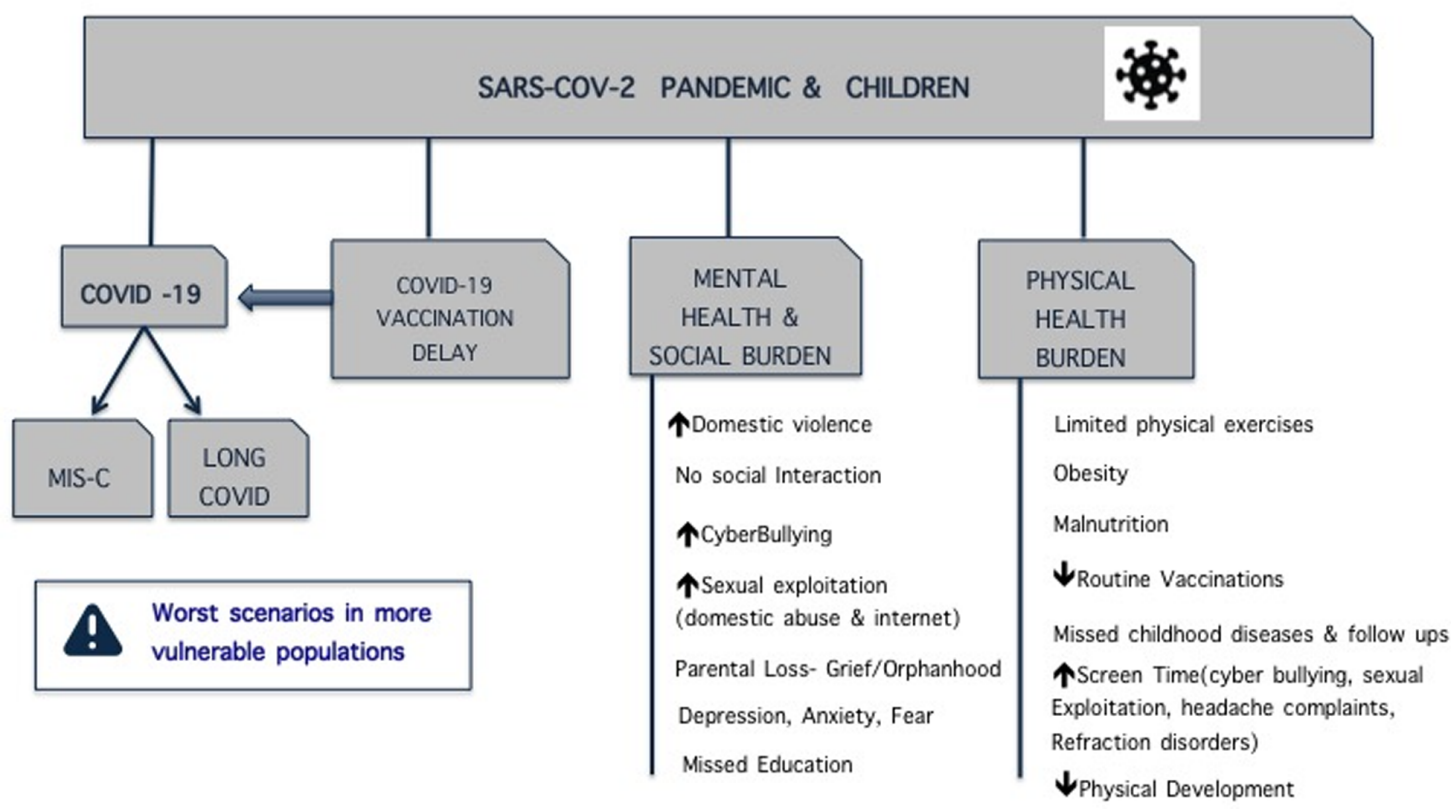
Effects of COVID-19 on children's health.

At the same time, although considered less at risk or “untouched” by the new virus, children had to pay the indirect price imposed by the new disease on the adult population, and possibly also by some wrong initial decisions. Risk of nosocomial transmission and public fear of in-hospital contagion led to delayed access to care, in many cases a reduction in elective surgeries (4), and restrictions for parent visits in hospitals, sometimes even in the PICU where the need for children (the patients) and their families to be together is at its highest (5). As with other decisions, the well-being of children was sacrificed for that of adults (Figure 1).

The extreme views regarding this discussion have had further negative consequences:

- Undermining the clinical burden of disease can create unrealistic expectations for families or diminish scientific interest and funds for the development of drugs or other necessary research/interventions focused on children.
- Exaggerating the clinical burden of COVID-19 in children creates unjustified fears which can lead to extreme, unnecessary actions that, in turn, can negatively affect the well-being of both children and families.

An example of a considered approach would be honest discussion around the real burden of children affected by Covid, regardless of them being either previously healthy or affected by other conditions. This leads to balanced decisions about which social measures to implement, balanced risk in schools, guidelines, and delays in care, and avoiding unnecessary closures and isolation of children, for example, in hospitals.

Long Covid entered later into the discussion. While it was quickly evident that a large subgroup of adults were not fully recovering after infection, this topic has been (and still is) much more debated in children. Observational studies of cohorts of children with COVID-19 without control groups, or studies including imperfect control groups (based on single negative PCR tests, or not fully sensitive serological studies), mostly based on phone calls or self-filled surveys, has led to heated debate (6). On one side is the incorrect argument that everything happening after COVID-19 is due to the infection itself; on the other, scientists have tried to undermine the problem by minimizing it as a psychologization of the condition, mainly due to the mental health impact of the restrictions rather than organic events. The debate is still raging, although scientific advances are starting to provide the rationale for the development of unexplained persisting symptoms in children. Observations of this problem throughout most of the world suggest that, at least for some families, pediatric Long Covid is a real problem.

Negative consequences from the extreme views in this discussion include:

- Reducing the problem to the idea that Long Covid is not a complication of the infection but a psychological problem. This leads to less research and funding in the field (slowing advances in terms of pathophysiology, diagnostics, and treatments), and inaccurate public risk perception of the overall burden of the SARS-CoV-2 infection in children. This can also make the families involved feel abandoned by healthcare systems.
- Conversely, exaggerating Long Covid creates inappropriate fear (which in turn can amplify the need for excessive restrictions), incorrect diagnoses, or late recognition of other treatable conditions. All of these can ultimately negatively affect children.

An example of a considered approach would be informing families about the benefit/risk ratio of vaccinations and other non-pharmacological interventions. It is important that families are made aware of all the possible complications of SARS-CoV-2 infection, from acute disease to post-covid complications, including MIS-C and Long Covid. Although the scientific community does not know yet the real incidence rate of Long Covid, there is agreement that at least a sub-group of children will develop it. Given the large number of undetected pediatric cases, it is possible that a low percentage of infected children will develop Long Covid, and luckily the pediatric disease seems less severe than the adult one. In any case, it is important that healthcare professionals take any child complaining of long-term clinical problems after Covid-19 seriously, rule out all possible alternative diagnoses with current available knowledge, and refer families to specialized centers. It is important for the family to receive care and that their children's conditions are not minimized.

One of the early responses to the sudden medical crisis was generalized lockdown, including school closures. This was initially justified by the massive number of sick adults filling hospitals, the still initially unknown impact of Covid-19 on children, and the possible role of children in viral transmission. However, while adult activities have been restored, in most of the world, school attendance was still being affected as of April 2022. Again, opposing arguments were raised. On one hand, the negative impact of school closures is evident: millions of children missed school, and most never came back, mainly in low- and middle-income countries (LMICs); children missed healthy school meals which, for struggling families, may represent an important source of nutrition; concerns regarding abuse or neglect of children living in difficult social environments were raised; there was an increase in mental health issues; and children with special needs were isolated at home for months if not years (7). On the other hand, certain groups actively argued there was a non-negligible risk for in-school transmission leading to acute Covid-19 or Long Covid (8). The distance between these positions led to indecision regarding difficult policies for in-school lessons that, in the best scenario, continued disrupting school attendance for months, or also led to unfeasible policies for LMICs. For two entire years, millions of children and adolescents have been considered cases, contacts, numbers, “positives or negatives”, and lost their right to be what they are supposed to be, that is, learners, or simply, children.

Linked to this topic is the “mask debate”. For some, while having a limited impact on covid transmissions, masks limit visual interaction and negatively affected neurocognitive development (9). For others, masks are a key solution and children of any age should be masked (8), even in the paradox that in many countries adults are not mandated to wear a mask yet 2-year-old children do.

Negative consequences from the extreme views in this discussion include:

- Schools being considered safe. That is, there is no need to improve current school statuses, therefore the chance to improve weak organizations or historical pre-covid problems is missed.
- Conversely, schools being seen as drivers of infection, leading to opinions asking for either a perfect solution or online learning, ultimately disrupting the whole school experience for years.

An example of a considered approach would be that schools represent a pivotal period in children's growth in terms of social, cultural, and physical relationships. The negative impact of indiscriminate school closures has become more evident than ever, while the key role of schools in significantly fueling the pandemic has never been proven. Therefore, school closures should only be considered in case of dramatic global or local scenarios, and in the context of generalized lockdowns, particularly during outbreaks that have a more significant impact on adults than on children. Importantly, school closures should only be employed for short periods and personalized programs for special needs children or those from very fragile social contexts should be implemented. At the same time, it is important to also recognize that several children living in a close environment may still be a risk scenario for transmission of airborne infections, and this pandemic should be taken as an opportunity to improve the prevention of airborne infections, as society has historically done with waterborne or foodborne infections. There is evidence that air cleaners may have a role in this regard and such a direction should be seriously considered (10). Additionally, there is no strong evidence either in favor of or against using masks, and therefore families should be informed of the risks and benefits and should be given the opportunity to choose whether to use them or not, according to their perception of the risk, community transmission, and child compliance.

Lastly, pediatric vaccinations have led to strong discussions mainly between, again, two different and distant factions: those in favor of or those against Covid-19 vaccinations. Those against vaccination highlight a non-irrelevant risk of myocarditis in young males and relatively fewer benefits in terms of prevention of complications as compared with adults, since the risk of severe disease in children is much lower. However, this approach does not take into account all the possible outcomes of infection, nor the possibility that families can translate doubts toward Covid-19 vaccinations to routine pediatric vaccinations (11). On the other side, the pro-vaccination group have undermined adverse events and exaggerated benefits, for example, in terms of the reduction of transmission which would have led also to better school attendance.

Negative consequences from the extreme views in this discussion include:

- Some countries only allowing vaccination in children aged 5–11 children at later phases of the pandemic without giving access to vaccines to families willing to be vaccinated. This generates distance between the public and national health systems. Highlighting only the negative effects of the vaccination and undermining all the short- and long-term outcomes of the infection does not give the public the opportunity to make an informed choice, and can reduce the overall favorable opinion of the general population towards all vaccinations.
- Exaggerating the benefits of vaccination increases the distances between the factions and impairs trust in science by those initially skeptical. Also, this can create false certainties and sense of security in vaccinated people.

An example of a considered approach would be to properly explain all the possible outcomes of SARS-CoV-2 infection in children, and highlight the risks of vaccination and what can be realistically expected from them. For example: setting targets for vaccinations, using realistic steps according to the main objectives of that particular period (e.g., deaths and hospitalizations in the early phases of a pandemic); and properly informing the public about the possible needs to adapt vaccine use as knowledge about new vaccines improve (in terms of safety, doses, goals, and technological implementation). This will allow families to make an informed choice with less risk of repercussion or criticism during rapidly changing scenarios.

We hope institutions, including scientific and societal ones, will develop a multilevel process for the critical analysis of the 2 years of the pandemic and develop appropriate plans for a balanced response to the next pandemic, taking into account the specific needs of children.
